# Correlation between frataxin expression and contractility revealed by in vitro Friedreich’s ataxia cardiac tissue models engineered from human pluripotent stem cells

**DOI:** 10.1186/s13287-019-1305-y

**Published:** 2019-07-08

**Authors:** Andy On-Tik Wong, Gabriel Wong, Michael Shen, Maggie Zi-Ying Chow, Wan Wai Tse, Bimal Gurung, Suet Yee Mak, Deborah K. Lieu, Kevin D. Costa, Camie W. Chan, Alain Martelli, Joseph F. Nabhan, Ronald A. Li

**Affiliations:** 1 Novoheart, Vancouver, British Columbia V6C 2V6 Canada; 2 Current address: Astellas Innovation Management Astellas Pharma, 1030 Massachusetts Avenue, Cambridge, MA 02138 USA

## Abstract

**Background:**

Friedreich’s ataxia (FRDA) is an autosomal recessive disease caused by a non-coding mutation in the first intron of the frataxin (*FXN*) gene that suppresses its expression. Compensatory hypertrophic cardiomyopathy, dilated cardiomyopathy, and conduction system abnormalities in FRDA lead to cardiomyocyte (CM) death and fibrosis, consequently resulting in heart failure and arrhythmias. Murine models have been developed to study disease pathology in the past two decades; however, differences between human and mouse physiology and metabolism have limited the relevance of animal studies in cardiac disease conditions. To bridge this gap, we aimed to generate species-specific, functional in vitro experimental models of FRDA using 2-dimensional (2D) and 3-dimensional (3D) engineered cardiac tissues from FXN-deficient human pluripotent stem cell-derived ventricular cardiomyocytes (hPSC-hvCMs) and to compare their contractile and electrophysiological properties with healthy tissue constructs.

**Methods:**

Healthy control and FRDA patient-specific hPSC-hvCMs were derived by directed differentiation using a small molecule-based protocol reported previously. We engineered the hvCMs into our established human ventricular cardiac tissue strip (hvCTS) and human ventricular cardiac anisotropic sheet (hvCAS) models, and functional assays were performed on days 7–17 post-tissue fabrication to assess the electrophysiology and contractility of FRDA patient-derived and FXN-knockdown engineered tissues, in comparison with healthy controls. To further validate the disease model, forced expression of FXN was induced in FXN-deficient tissues to test if disease phenotypes could be rescued.

**Results:**

Here, we report for the first time the generation of human engineered tissue models of FRDA cardiomyopathy from hPSCs: FXN-deficient hvCTS displayed attenuated developed forces (by 70–80%) compared to healthy controls. High-resolution optical mapping of hvCAS with reduced FXN expression also revealed electrophysiological defects consistent with clinical observations, including action potential duration prolongation and maximum capture frequency reduction. Interestingly, a clear positive correlation between FXN expression and contractility was observed (*ρ* > 0.9), and restoration of FXN protein levels by lentiviral transduction rescued contractility defects in FXN-deficient hvCTS.

**Conclusions:**

We conclude that human-based in vitro cardiac tissue models of FRDA provide a translational, disease-relevant biomimetic platform for the evaluation of novel therapeutics and to provide insight into FRDA disease progression.

**Electronic supplementary material:**

The online version of this article (10.1186/s13287-019-1305-y) contains supplementary material, which is available to authorized users.

## Background

Friedreich’s ataxia (FRDA) is a hereditary neuromuscular degenerative disease caused by a trinucleotide repeat expansion mutation in the first intron of the frataxin (*FXN*) gene [1]. Although FXN, a mitochondrial protein involved in the biosynthesis of iron-sulfur co-factors required by multiple mitochondrial and extra-mitochondrial proteins, is fully functional in FRDA patients, the intronic mutation significantly reduces the expression to only 5 to 30% of non-carriers [2, 3]. Clinically, the heart is a major site of pathology in FRDA: cardiac symptoms are first diagnosed as an abnormal electrocardiogram (EKG) and can progress to compensatory hypertrophic cardiomyopathy and dilated cardiomyopathy, followed by cardiomyocyte (CM) death and fibrosis, ultimately leading to arrhythmias and heart failure [4–7]. For FRDA patients, cardiac dysfunction is the leading cause of death [8].

With advances in reprogramming, human induced pluripotent stem cells (hiPSCs) have been derived from FRDA patients and differentiated into CMs to study the disease progression in a human in vitro model. Previous single-cell studies using FRDA hiPSC-derived CMs have demonstrated cellular phenotypes such as hypertrophy, accumulation of intracellular iron and reactive oxygen species (ROS), attenuated ATP production, and compromised Ca^2+^ handling consistent with patient symptoms [9–12]. However, considering that the heart is a 3-dimensional (3D) dynamic organ whose primary function is to pump, no multicellular model has been reported to study FRDA disease phenotypes at the tissue level, such as the impact on contractility. It is generally acknowledged that observations from 2-dimensional (2D) biological assays do not necessarily translate to 3D systems [13, 14], and 3D cardiac engineered tissues have proven valuable in modeling cardiac diseases including hypertrophic cardiomyopathy [15, 16]. Here, we investigated for the first time the effects of reduced FXN level on cardiac electrophysiological and contractile properties with various engineered tissue constructs fabricated from normal and FXN-deficient human embryonic stem cell (hESC)- and induced pluripotent stem cell (hiPSC)-derived ventricular cardiomyocytes (hvCMs). Specifically, our advanced engineered tissue platform permits generation of human ventricular cardiac anisotropic sheets (hvCAS), monolayers of strategically aligned hvCMs to reproduce structural and functional anisotropy of the native ventricle [17–19]. Using hvCAS, we measured cellular action potential parameters and electrical conduction as a syncytium, and with 3D human ventricular cardiac tissue strips (hvCTS) that resemble cardiac trabecular muscles [16, 20, 21], we evaluated contractility. Collectively, these hvCAS and hvCTS constructs offer a cardiomimetic in vitro model system which yields multicellular readouts such as conduction velocity and magnitude of force generation that are otherwise not possible in single-cell models. Our results are discussed in relation to their mechanistic and translational insights into FRDA.

## Methods

### hPSC culture and differentiation into CMs

FRDA patient-specific hiPSCs (FRDA(68) and FRDA(03665)) were obtained from the University of Alabama at Birmingham. Healthy hESCs (HES2; ESI, NIH code ES02) and FRDA(03665) were cultured on hESC-qualified Matrigel (Corning) with mTeSR1 medium (Stem Cell Technologies). Control hiPSCs (PB02), reprogrammed from peripheral blood mononuclear cells by episomal nucleofection of transcription factors—OCT3/4, SOX2, KLF4, L-MYC, and LIN28—plus p53-interfering shRNA, and FRDA(68) were cultured on hESC-qualified Geltrex (Gibco) with Essential 8 Medium (Gibco). All hPSC lines were cultured at 37 °C with 5% CO_2_. To differentiate hPSCs into cardiomyocytes, dissociated hPSCs were allowed to form cell clusters in either mTeSR1 or Essential 8 (same as in regular culture) with Matrigel and 1 ng/ml bone morphogenetic protein 4 (BMP4) overnight in an ultra-low attachment plate and hypoxic condition. From day 1 to 4, cell clusters were treated with 50 μg/ml ascorbic acid (Sigma-Aldrich), 10 ng/ml activin A, 10 ng/ml BMP4, and 10 μM ROCK inhibitor Y-27632 in StemPro-34 medium supplemented by GlutaMAX (Thermo Fisher Scientific) in hypoxic condition. Next, cell clusters in hypoxic condition were treated with 50 μg/ml ascorbic acid and 5 mM IWR-1 in StemPro-34 medium until day 8. Cell clusters were maintained after day 8 in normoxic condition with StemPro-34 media containing 50 μg/ml ascorbic acid. Using this differentiation protocol [22], ventricular subtype yield of over 70% of hPSC-derived CMs was achieved. These differentiated cells are referred to as human ventricular (hv) CM and were used in all experiments presented in this study. Differentiation yield from hESC and hiPSC lines in this study had a median of 73–77%, as estimated by the percentage of cardiac troponin T-positive cells (Additional file 1: Figure S1).

### Knockdown and overexpression of FXN in hvCMs

**Fig. 1:**
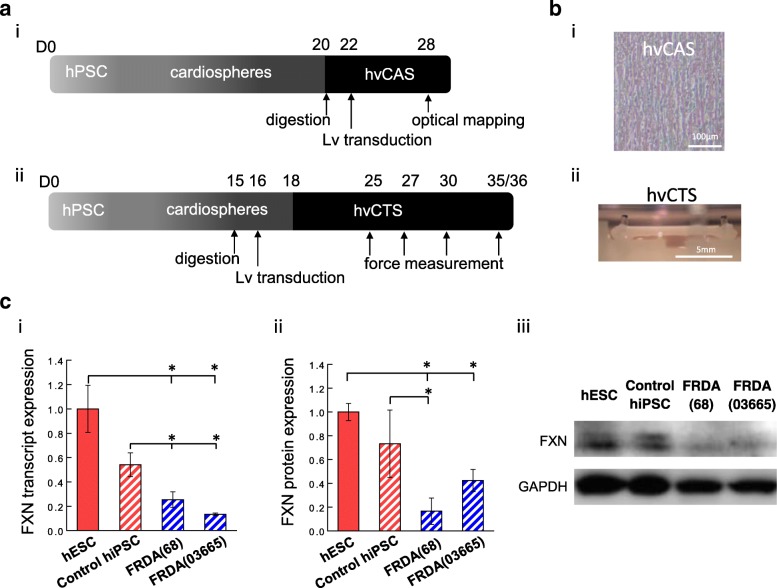
In vitro modeling of FRDA by engineered cardiac tissue constructs of FXN-deficient hPSCs. **a** Experimental timeline for generating cardiac tissue models, human ventricular cardiac anisotropic sheet (hvCAS), and human ventricular cardiac tissue strip (hvCTS), for electrophysiological and contractile assessment, respectively. **b** Representative images of (i) hvCAS and (ii) hvCTS. **c** FXN (i) transcript and (ii) protein expression and (iii) representative Western blot image of hESCs (*n* = 8–9), hiPSCs (*n* = 5–9), and FRDA-hiPSC lines 68 (*n* = 8–10) and 03665 (*n* = 3–4), normalized to GAPDH expression. Samples were independent replicates. Data are shown as mean ± SEM. Statistical significance indicated by *p < 0.05

To model FXN deficiency of FRDA in hESC- and hiPSC-derived hvCMs, FXN was knocked down in both types of hvCMs by transduction with lentiviral shRNA (Lv-shFXN1: TRCN0000006137 or Lv-shFXN2: TRCN0000010996 inserts in pLKO.1 vector backbone) at MOI of 5 following the timeline in Fig. 1a. Respective control hvCMs were transduced with mammalian non-targeting shRNA (Lv-shNT). To restore FXN expression, FXN-deficient hvCMs were transduced with lentivirus to overexpress FXN (Lv-FXN; GE Dharmacon OHS5835-EG2395), with lentivirus delivering red fluorescent protein (Lv-RFP) serving as a control. To assess and compare the electrophysiological and contractile functions among healthy, FXN-deficient, and/or FXN-overexpressing hvCMs, two tissue construct platforms were constructed from these cells, each specifically designed to enable functional assessment as described below. Control hiPSC groups were not virally transduced except for where specified otherwise.

### Contractile assessment of human ventricular cardiac tissue strips

To assess hvCM contractile function by direct measurement of contractile force generation, 3D multicellular hvCTS myocardial tissues were engineered as previously described [20, 21]. Briefly, cardio-clusters from day 15 of hPSC cardiac differentiation were dissociated into single cells and allowed to recover in the incubator for 3 days before hvCTS construction. Each hvCTS consisted of 1.3 × 10^6^ cardiac cells differentiated from hPSCs and 1.3 × 10^5^ human foreskin fibroblasts in a 100-μl ice-cold solution of 2 mg/ml collagen I (Thermo Fisher Scientific), 0.80–0.95 mg/ml Matrigel, 0.6× PBS, 20 mM NaOH, 0.8× Minimum Essential Medium (Sigma-Aldrich), 1.6 mM HEPES, and 0.1× hvCTS maintenance medium (see composition below). A volume of 100 μl of the final cell-collagen mixture is then added to each polydimethylsiloxane (PDMS) bioreactor, consisting of a force-sensing cantilever post at each end of a rectangular well, and returned to the incubator to form the hvCTS attached between the two end posts. The hvCTS were maintained in DMEM medium supplemented with 10% newborn calf serum (Gibco), with daily half-medium changes, until ready for testing.

Force generated by the hvCTS was measured at 37 °C in phenol red-free DMEM medium with HEPES buffer using either a custom-designed post-tracking force measurement system that records displacement of the cantilever posts on a temperature-controlled heating plate, or in a commercial isometric muscle bath system (Aurora Scientific) at the end of the study, as specified in the “Results” section. During testing, the hvCTS were paced by electrical field stimulation at 1 Hz frequency. An overview of the experimental timeline is shown in Fig. 1a (ii). For longitudinal studies, the hvCTS were returned to the incubator with fresh media following testing at intermediate timepoints.

### Electrophysiological assessment of human ventricular cardiac anisotropic sheets

To assess the electrophysiological properties of hvCMs as an aligned and electrically coupled syncytium with anisotropic conduction property similar to ventricular CMs in vivo, cardio-clusters from day 20 of hPSC cardiac differentiation were dissociated into single cells, and 4.5 × 10^5^ cells/cm^2^ were plated as a monolayer on Matrigel-coated, microgrooved substrates fabricated from polystyrene shrink film (Shrinky Dinks “Crystal Clear,” K&B Innovations), with groove width of 15 μm, groove depth of 5 μm, and inter-groove distance of 5 μm, to form hvCAS as previously described [18]. After allowing recovery for 8 days, action potentials of hvCAS were optically mapped with a MiCAM ULTIMA imaging system (SciMedia) using voltage-sensitive fluorophore Di-8-ANEPPS with Pluronic F-127 (Thermo Fisher Scientific) in Tyrode’s solution containing blebbistatin (Sigma-Aldrich). Automaticity, threshold voltage, and maximum capture frequency (MCF) were first determined for each hvCAS, followed by programmed electrical stimulation to test for reentrant arrhythmias. An overview of the experimental timeline is shown in Fig. 1a (i).

## Results

### FRDA hiPSCs exhibited reduced FXN expression

To create human engineered cardiac tissue models of FRDA, hPSCs were differentiated into hvCMs and fabricated into hvCAS and hvCTS, following a defined schedule (Fig. 1a, b). FXN expression was first assessed in four hPSC lines, including one healthy hESC, one healthy hiPSC, and two FRDA-hiPSC lines—FRDA(68) and FRDA(03665)—reprogrammed from two FRDA patients. FXN at the transcript level was comparable for the two FRDA-hiPSC lines, with both expressing < 50% of the FXN levels observed in the healthy hESC and hiPSC lines (Fig. 1c (i)). The protein level for FXN was in agreement with the transcripts, with FRDA-hiPSCs expressing the lowest level of FXN relative to the healthy hPSC lines (Fig. 1c (ii, iii)).

### Isogenic FXN-knockdown models of hESC-hvCMs exhibited reduced FXN expression and contractile function

**Fig. 2:**
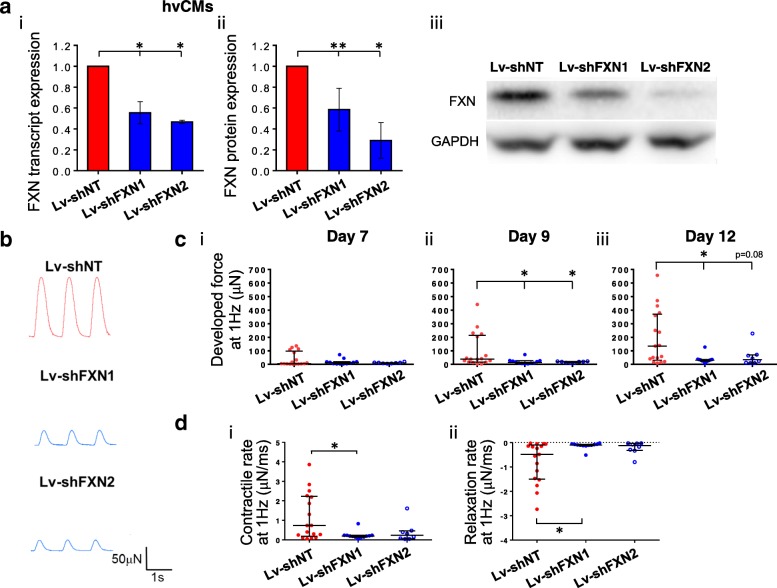
Isogenic FRDA cardiac model derived from hESCs. **a** FXN (i) transcript and (ii) protein expression (normalized to GAPDH expression) and (iii) representative Western blot image of hESC-hvCMs transduced with Lv-shFXN1 and Lv-shFXN2, relative to batch-matched control transduced with Lv-shNT (*n* = 3–6). Transcript and protein data were generated from 3 to 6 and 3 to 5 independent differentiation batches, respectively. Data are shown as mean ± SEM. **b** Representative contractile force traces on day 12 for hESC-hvCTS transduced with Lv-shFXN1 and Lv-shFXN2, with Lv-shNT as control. **c** Developed force generation on (i) day 7, (ii) day 9, and (iii) day 12 from 1 Hz-paced hESC-hvCTS transduced with Lv-shFXN1 (*n* = 12) and Lv-shFXN2 (*n* = 8), compared to control transduced with Lv-shNT (*n* = 17). hESC-hvCTS force generation data originated from 3 to 6 independent batches of differentiation. **d** Kinetics analysis of (i) contractile rate and (ii) relaxation rate of hESC-hvCTS force generation on day 12. All force generation data are shown as median with interquartile range. Statistical significance indicated by *p < 0.05 and **p < 0.01

To model FRDA in isogenic human CMs in vitro, hESC-derived hvCMs were transduced with the lentiviral construct Lv-shFXN1 or Lv-shFXN2 for shRNA-mediated FXN suppression or with non-targeting Lv-shNT as control. Both Lv-shFXNs successfully induced FXN knockdown at the transcript level, as demonstrated by ~ 50% reduced transcript expression in hESC-vCMs relative to Lv-shNT-transduced control (Fig. 2a (i)). The reduction in FXN protein in hvCMs was 40% and 70% for Lv-shFXN1 and Lv-shFXN2, respectively (Fig. 2a (ii, iii)). Using these transduced hESC-hvCMs to construct hvCTS, contractility was then assessed as developed force generation during electrical field stimulation at 1 Hz on days 7, 9, and 12 after tissue fabrication (Fig. 2b, c). Contractile force in control hESC-hvCTS progressively increased from a median of 5 μN on day 7 to 135 μN on day 12. Conversely, hESC-hvCTS with FXN knockdown by either Lv-shFXN construct failed to demonstrate a significant increase in developed force over time. By day 12, the median force in FXN-deficient hvCTS was 75–80% lower than that of control (Fig. 2c (iii)). In addition, the contraction and relaxation rates were > 3 times slower in FXN-deficient hvCTS (Fig. 2d). There was no significant difference in developed force between non-transduced and Lv-shNT hESC-hvCTS (data not shown).

### Patient-derived FRDA cardiac models were FXN-deficient with compromised contractility

**Fig. 3:**
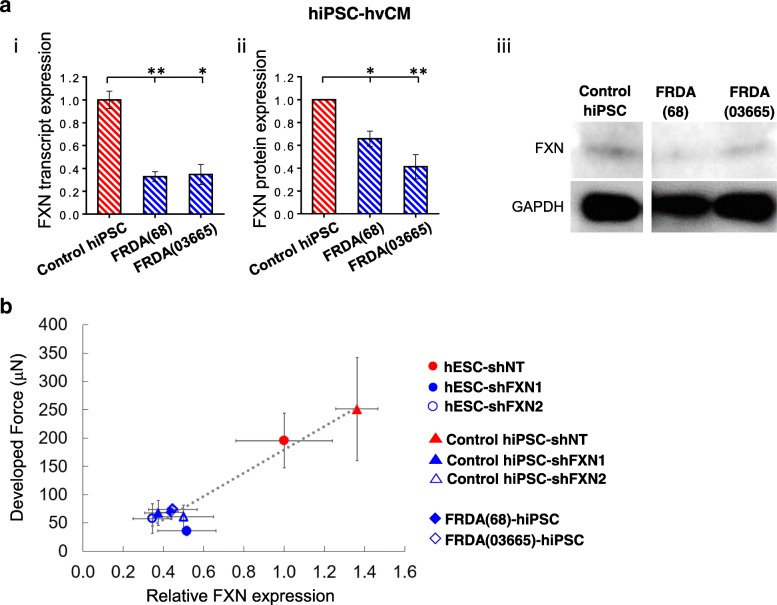
FRDA cardiac model derived from FRDA-hiPSCs. **a** FXN (i) transcript and (ii) protein expression (normalized to GAPDH expression) and (iii) representative Western blot image of FRDA(68)-hiPSC-hvCMs and FRDA(03665)-hiPSC-hvCMs relative to healthy control hiPSC-hvCMs (*n* = 3–5). Transcript and protein data were generated by 3–4 and 3–5 independent differentiations, respectively. **b** Correlation of developed force to FXN transcript expression in hESC- and hiPSC-hvCTS transduced with Lv-shNT, Lv-shFXN1, Lv-shFXN2, and FRDA-hiPSC-hvCTS relative to the hESC-shNT group (on day 12, at 1 Hz pacing). Force generation and FXN transcript expression data were generated from 3 to 8 and 3 to 6 independent cell differentiation batches, respectively. Data are shown as mean ± SEM. Statistical significance indicated by *p < 0.05 and **p < 0.01

FXN expression was assessed at both the transcript and protein levels for hvCMs derived from FRDA(68)-hiPSCs, FRDA(03665)-hiPSCs, and healthy hiPSCs. hvCMs differentiated from FRDA(68)-hiPSCs and FRDA(03665)-hiPSCs expressed about 70% less FXN at the transcript level (Fig. 3a (i)), and nearly 40–60% less at the protein level, compared to controls from healthy donors (Fig. 3a (ii, iii)). Force was measured after the construction of FRDA-hiPSC-hvCMs into hvCTS. To correlate the force generation to FXN expression, the developed forces in day 12 hvCTS at 1 Hz of all groups recorded were plotted against the FXN transcript levels (Fig. 3b). Generally, FXN-deficient hvCTS—due to either shRNA-mediated knockdown or intrinsic genetic defect in FRDA-hiPSC—exhibited compromised force generation by developing less than half of the force as healthy controls that expressed robust FXN. As indicated by Pearson’s coefficient of 0.96, there was a strong correlation between the levels of developed force and FXN expression.

### Electrophysiological properties were altered in FXN-deficient hvCAS

**Fig. 4:**
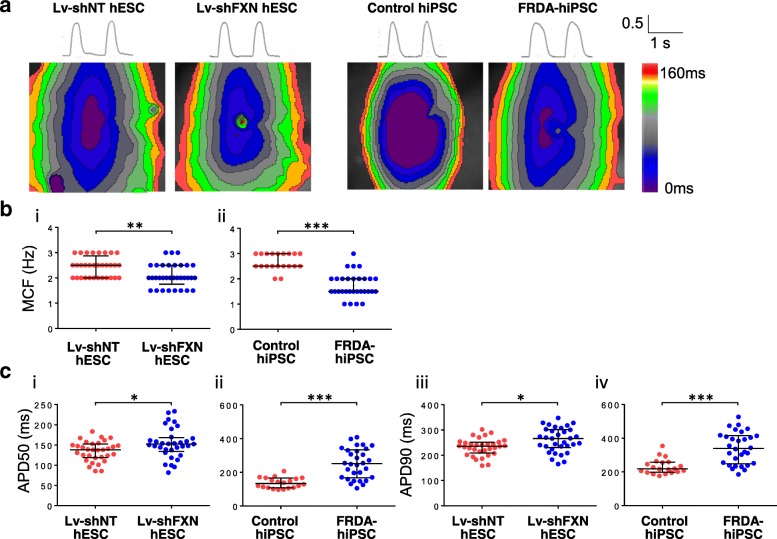
Electrophysiological measurements from FXN-deficient hvCAS derived from hESCs and hiPSCs relative to their respective healthy controls. **a** Representative action potentials and isochronal maps from control hESC-hvCAS transduced with non-targeting lentivirus (Lv-shNT), hESC-hvCAS transduced with Lv-shFXN, control hiPSC-hvCAS, and FRDA-hiPSC-hvCAS. **b** Maximum capture frequency (MCF) for (i) Lv-shNT (*n* = 32) vs. Lv-shFXN (*n* = 33) hESC-hvCAS and (ii) healthy control hiPSC-hvCAS (*n* = 20) vs. FRDA-hiPSC-hvCAS (*n* = 30). **c** Action potential duration at 50% repolarization (APD50) and 90% repolarization (APD90) derived from optical mapping of (i and iii) Lv-shNT vs. Lv-shFXN-transduced hESC-hvCAS and (ii and iv) healthy control vs. FRDA-hiPSC-hvCAS. Data were generated from 3 to 9 independent batches of differentiation. All electrophysiology data are shown as median with interquartile range. Statistical significance indicated by *p < 0.05, **p < 0.01, and ***p < 0.001

Next, the electrophysiological properties of shRNA-suppressed and FRDA hPSC-derived hvCMs were assessed by high-resolution optical mapping of hvCAS in comparison with the corresponding controls (Fig. 4a). The incidence of spiral conduction waves and automaticity (i.e., spontaneous generation of action potentials) were not statistically different between hESC-hvCAS transduced with Lv-shFXN and LV-shNT-transduced control (Additional file 1: Table S1). However, the MCF of Lv-shFXN-transduced hESC-hvCAS, with a median of 2.0 Hz, was statistically lower than control (2.5 Hz; Fig. 4b (i)). Similarly, FRDA-hiPSC-derived hvCAS were harder to capture at higher pacing frequency compared to control (median MCF = 1.5 Hz for FRDA-hiPSC-hvCAS vs. 2.5 Hz for healthy hiPSC-hvCAS; Fig. 4b (ii)).

Action potential and conduction parameters revealed further electrophysiological differences between healthy and FXN-deficient hvCAS (Fig. 4c). Action potential upstroke and decay velocities and anisotropic ratio did not show consistent differences between FXN-deficient hvCAS and control for both the hESC and hiPSC groups (Additional file 1: Table S1). However, action potential duration at 50% repolarization (APD50) and 90% repolarization (APD90) were significantly prolonged in hESC-hvCAS transduced with Lv-shFXN (APD50, 152 ms; APD90, 266 ms) relative to control (APD50, 138 ms; APD90, 236 ms) (Fig. 4c (i, iii)). Notably, APD50 and APD90 were similarly prolonged in FRDA-hiPSC-hvCAS compared to healthy controls (Fig. 4c (ii, iv)).

### Restoration of FXN expression rescued hvCTS contractile function

**Fig. 5:**
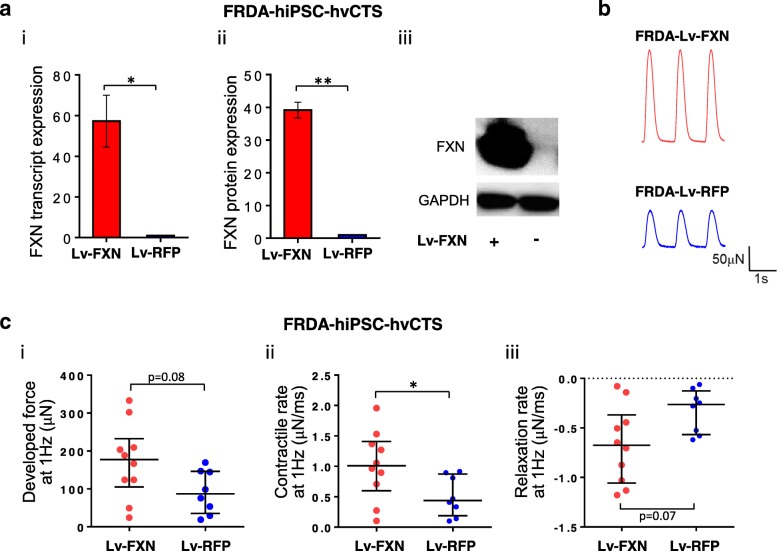
FXN expression rescue of FRDA hiPSC-hvCTS model. **a** FXN (i) transcript and (ii) protein expression (normalized to GAPDH expression) and (iii) representative Western blot image of FRDA-hiPSC-hvCTS after Lv-FXN transduction relative to Lv-RFP control (*n* = 3–4). Transcript and protein data were generated from 3 to 4 independent differentiation batches, respectively. **b** Representative contractile force traces on days 17–18 for FRDA-hiPSC-hvCTS transduced with Lv-FXN with Lv-RFP as control. **c** (i) Developed force, (ii) contractile rate, and (iii) relaxation rate generated at 1 Hz pacing in FRDA-hiPSC-hvCTS transduced with Lv-FXN (*n* = 10) relative to Lv-RFP control (*n* = 8). Data were generated by 4 independent batches of cell differentiation. FXN expression data are shown as mean ± SEM, and force generation data are shown as median with interquartile range. Statistical significance indicated by *p < 0.05 and **p < 0.01

Considering the strong positive correlation between the developed force and FXN expression, we postulated that restoration of FXN expression would suffice to rescue the compromised contractile function in the FXN-deficient hvCTS. To test this, FXN expression was rescued in FRDA-hvCTS by transduction with Lv-FXN. FXN deficiency in FRDA-hvCTS transduced with Lv-FXN was rectified as indicated by ~ 60-fold and ~ 40-fold higher expression of FXN transcript and protein, respectively, compared to control transduced with Lv-RFP (Fig. 5a). Contractile force developed at 1 Hz pacing in Lv-FXN-transduced FRDA-hvCTS on days 17–18 post-construction was elevated, with a median of 178 μN compared to control of 87 μN (Fig. 5b, c (i)). In addition, the contraction and relaxation rates were increased by ~ 3-fold in the Lv-FXN group compared to Lv-RFP control (Fig. 5c (ii, iii)).

**Fig. 6:**
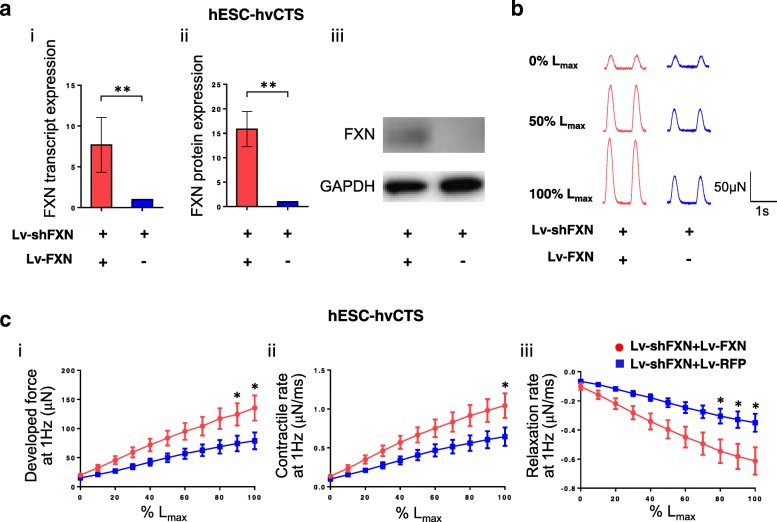
FXN expression rescue of FRDA hESC-hvCTS model. **a** FXN (i) transcript and (ii) protein expression (normalized to GAPDH expression) and (iii) representative Western blot image of hESC-hvCTS double-transduced with Lv-shFXN and Lv-FXN relative to Lv-shFXN and Lv-RFP control (*n* = 5–6). Transcript and protein data were generated from 5 to 6 independent batches of cell differentiation, respectively. **b** Representative contractile force traces from isometric force measurement relative to % *L*_max_ for hESC-hvCTS double-transduced with Lv-shFXN and Lv-FXN, relative to Lv-shFXN and Lv-RFP control. **c** Developed force, contractile rate, and relaxation rate for % *L*_max_ measured at 1 Hz stimulation using isometric force measurement for hESC-hvCTS double-transduced with Lv-shFXN and Lv-FXN, relative to Lv-shFXN and Lv-RFP control (Lv-shFXN+Lv-FXN: *n* = 16; Lv-shFXN+Lv-RFP: *n* = 11). Data were generated by 4 independent differentiation batches. Data are shown as mean ± SEM. Statistical significance indicated by *p < 0.05 and **p < 0.01

We next tested the functional consequences of restored FXN expression in the complementary isogenic hESC-hvCTS FRDA model—Lv-shFXN+Lv-FXN group for simultaneous knockdown and forced expression of FXN and Lv-shFXN+Lv-RFP group with FXN knockdown and RFP control. As anticipated, FXN transcript and protein expression were restored for hESC-hvCTS transduced with Lv-shFXN+Lv-FXN, at levels significantly higher than in Lv-shFXN+Lv-RFP-transduced control (Fig. 6a). Further isometric force measurements, using a physiologic muscle bath to stretch the hvCTS length in 0.225 mm (2.5% strain) increments on day 18 post-construction, revealed that the developed force, under 1 Hz pacing, increased with increasing muscle strip length in both hvCTS groups, reaching the maximum contractile force at a length defined as *L*_max_ (Fig. 6b). Similar to the FXN restoration-induced effects observed in the FRDA-hiPSC model, the isometric developed force was significantly higher in the Lv-shFXN+Lv-FXN hESC-hvCTS FRDA model at strains greater than 90% *L*_max_ (physiological range of ~ 17.5–27.5% strain) compared to the control group (Fig. 6 c (i)). At *L*_max_, hESC-hvCTS rescued by FXN expression exhibited a mean force of 135 μN compared to the FXN-deficient control at 79 μN. The force development kinetics were also significantly faster in the hESC-hvCTS FRDA model with restored FXN expression, showing a mean contraction rate of 1.04 μN/ms vs. control of 0.64 μN/ms at *L*_max_ and a mean relaxation rate of − 0.61 μN/ms vs. control of − 0.35 μN/ms at *L*_max_ (Fig. 6c (ii, iii)).

## Discussion

Heart failure and arrhythmia are the major causes of mortality in FRDA patients [4–7]. Given the high level of amino acid identity among the *FXN* genes across species, murine models have been generated for studying FRDA. One murine model based on conditional ablation of mouse *Fxn* results in complete knockout of *Fxn* in cardiac and skeletal muscle [23]; although this model presents an aggressive FRDA-like phenotype, including cardiomyopathy and mitochondrial defects, it does not mirror the human genotype of trinucleotide repeats, which partially silence expression of the otherwise fully functional protein. Several other mouse models have been generated through transgenic expression of human *FXN* with expanded GAA repeats, knock-in of a GAA repeat expansion into the mouse *Fxn* locus, or by shRNA knockdown of mouse FXN expression, with variable severities of the resultant phenotype [24–26]. Since the use of human models may be more clinically relevant, in the present study we generated engineered human cardiac models of FRDA hvCTS and hvCAS to enable robust in vitro phenotypic readouts.

Despite different genetic backgrounds, similar contractile (weakened developed force) and electrophysiological (reduced MCF and APD prolongation) phenotypes were observed in engineered constructs made out of isogenic hESCs by FXN knockdown or reprogrammed FRDA-specific hiPSCs in which FXN expression is suppressed by pathological GAA expansions. The results suggested a general important role of FXN in cardiobiology. Indeed, a strong positive correlation between developed force and FXN expression was revealed by the collective data of various normal and FRDA hESC/iPSC-hvCTS models. Considering that cardiomyocytes contain the highest mitochondrial density to sustain energy requirement [27] and that FXN promotes the biosynthesis of iron-sulfur clusters, which are co-factors required by mitochondrial proteins such as aconitase and succinate dehydrogenase, reduced levels of FXN are likely to yield adverse effects on mitochondrial function and thereby compromise contractility. Consistently, the attenuated developed force in FRDA hvCTS could be rescued via FXN overexpression. This is in accordance with the inducible and reversible murine FRDA model that demonstrated FXN expression can reverse pathological effects [26]. The exact mechanism that is responsible for the observed disease phenotype and rescue of pathological effects by FXN expression in our human cardiac tissue constructs will need further investigation and may provide novel insights for therapeutic strategies.

Moreover, our work is the only study to date that demonstrated electrophysiological and contractile dysfunction from functional assays comparing healthy and FRDA (FXN-deficient) hPSC models. Using bright-field time-lapse imaging, beating rate and incidence of arrhythmic activity of control and patient hiPSC-derived cardiomyocytes were previously studied; however, functional data were not reported [9]. Although two publications by Lee et al. [10, 11] examined Ca^2+^-handling properties, no significant differences in Ca^2+^ transient amplitude and rate of rise or decay were observed between the healthy and FRDA-hiPSC-derived cardiomyocytes. To investigate the efficacy of drugs in clinical trials for treating FRDA, Lee et al. induced pathological effects with the addition of 200 μM Fe^2+^ to both the FRDA and healthy hiPSC-derived cardiomyocyte culture. A difference in Ca^2+^ amplitude and rate of decay between the healthy and FRDA groups was only revealed under such intervention. However, physiological relevance is a concern since healthy cardiomyocytes would not progress to such an iron-overloaded state. Likewise, the results of drug treatments that chelate iron, when tested on iron-overloaded healthy and FRDA hiPSC-cardiomyocytes, may not be predictive of drug efficacy for clinical treatment of FRDA. Therefore, the engineered tissues used in this report without exogenous factors to induce pathological conditions may offer advantages as physiologically relevant models for investigating pathological effects stemming from FXN deficiency and for therapeutic testing.

Electrophysiologically, FXN-deficient hvCAS exhibited action potential prolongation or repolarization delay. The median APD90 prolongation due to FXN deficiency was 30 and 110 ms in isogenic hESC- and hiPSC-hvCAS models, respectively. These effects are physiologically relevant, as a prolongation of 20 ms or more in the QT interval is considered to be a high risk for torsade de pointes under FDA guidelines. This observation was also in agreement with T-wave inversion or abnormal repolarization of the ventricle recorded in the EKG of FRDA patients that precede the presentation of FRDA cardiomyopathy [7]. Hence, our in vitro human FRDA cardiac tissue model was able to replicate early pathology associated with disease-related cardiomyopathy.

## Conclusions

In summary, we demonstrated that the clinical symptoms of contractile and electrophysiological dysfunction in FRDA patients can be recapitulated by human cardiac tissues engineered from FXN-deficient hPSC-derived hvCMs. Translationally, the positive correlation between FXN expression and contractility and the results of our rescue experiments underscore the potential of FXN restoration by small molecules or gene therapy as an effective therapeutic strategy for suppressing or even reversing the cardiac symptoms of FRDA.

## Additional file

Additional file 1: **Figure S1.** Percentage yield of cardiomyocytes differentiated from various hESC and hiPSC cell lines. **Table S1.** Electrophysiological parameters of hvCAS. (PDF 145 kb)

## Data Availability

All supporting data are included in the article and its additional files.

## Abbreviations

FRDA: Friedreich’s ataxia
FXN: Frataxin
hESCs: Human embryonic stem cells
hiPSCs: Human induced pluripotent stem cells
hPSCs: Human pluripotent stem cells
hvCAS: Human ventricular cardiac anisotropic sheets
hvCMs: Human ventricular cardiomyocytes
hvCTS: Human ventricular cardiac tissue strips
