# Supplementary material for: Correlation between frataxin expression and contractility revealed by in vitro Friedreich’s ataxia cardiac tissue models engineered from human pluripotent stem cells
Source: Stem Cell Res Ther. 2019 Jul 8;10:203. doi: 10.1186/s13287-019-1305-y (PMC6615274; doi:10.1186/s13287-019-1305-y)
Supplement: Supplementary file 1 — Figure S1. Percentage yield of cardiomyocytes differentiated from various hESC and hiPSC cell lines. Table S1. Electrophysiological parameters of hvCAS. (PDF 145 kb) [file 13287_2019_1305_MOESM1_ESM.pdf]

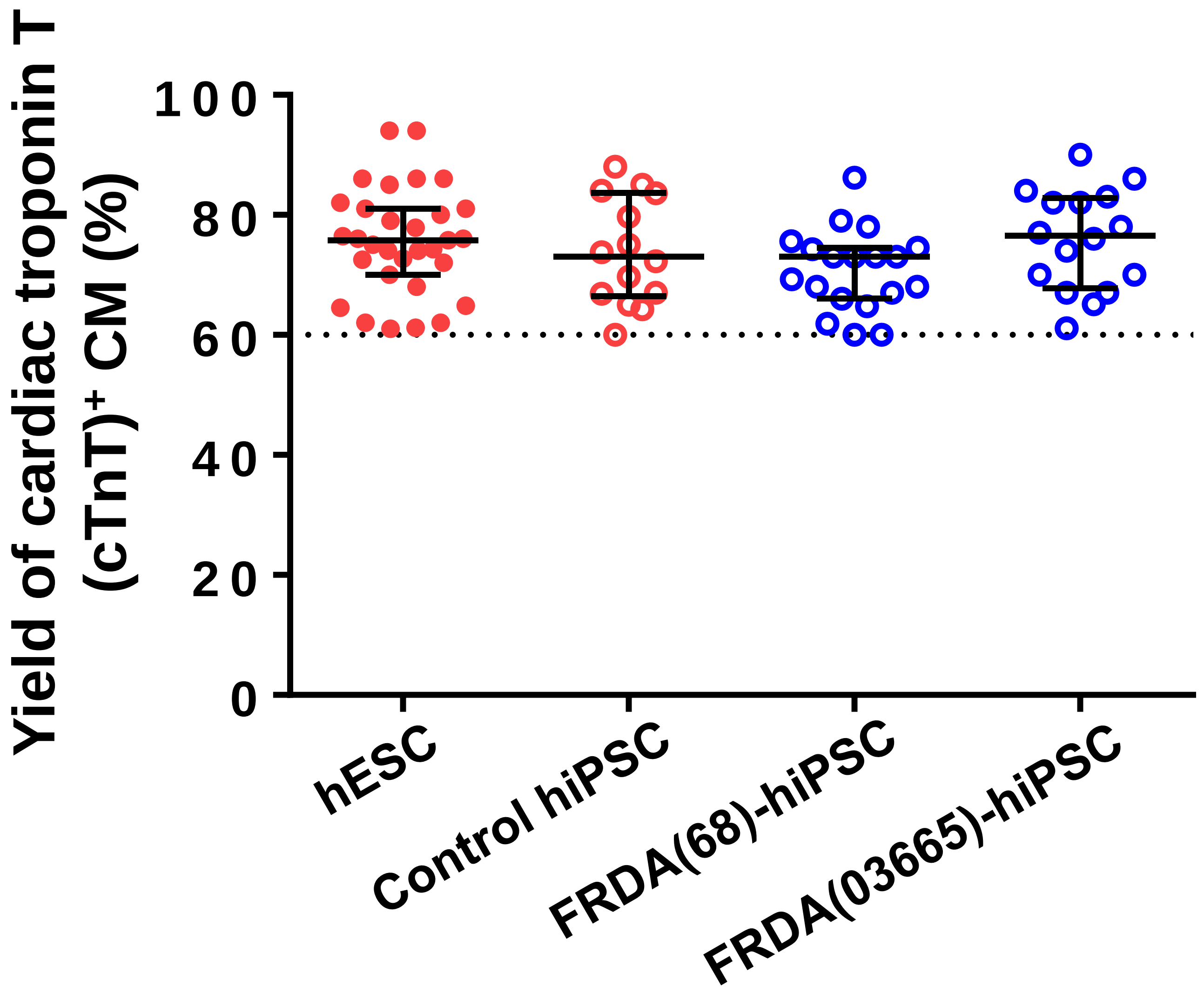

Supplemental Figure 1

		Lv-shNT hESC	Lv-shFXN hESC	Control hiPSC	FRDA hiPSC
Upstroke velocity	Fluorescence intensity change/ time (ΔF/ms)	0.023 ± 0.001	0.018 ± 0.002	0.032 ± 0.002	0.038 ± 0.003
Decay velocity		-0.016 ± 0.001	-0.012 ± 0.001	-0.019 ± 0.001	-0.020 ± 0.001
Longitudinal conduction velocity	cm/s	6.32 ± 0.35	5.17 ± 0.30	2.76 ± 0.23	9.10 ± 0.69
Transverse conduction velocity		2.77 ± 0.15	2.16 ± 0.16	0.95 ± 0.03	4.32 ± 0.39
Anisotropic ratio		2.49 ± 0.14	2.62 ± 0.14	2.95 ± 0.26	2.40 ± 0.16
Preparations with spontaneous action potential firing		3.1 %	18.2 %	60.0 %	13.3 %
Preparations with spiral reentrance		6.3 %	9.1 %	40.0 %	3.3 %

Supplemental Table 1
